# The Treatment of Syphilis

**Published:** 1872-11

**Authors:** M. P. Ricord

**Affiliations:** Paris


### The Treatment of Syphilis.

By M. P. RICORD, Paris.

I must thank you very much for the reception you have accorded to me, and I am deeply gratified at finding that my name and reputation are known to you. I have not prepared anything to say, and I did not intend to speak; but Mr. Acton caught me, and has forced me to speak. It is a trick. I came here to listen, and not to speak—to learn and not to teach; but, if I am to speak, it will be little, after what Mr. Acton has said; for he has entirely given my views on the phases of syphilis, on the peculiarity of symptoms, and on the manner of treatment.

There is one question which comes before the medical man very frequently: Can syphilis be cured radically? That is the question which we will consider. There is an immense quantity of venereal disease cured—clap, swelling of the glands, soft chancres, warts—all these "accidents," not belonging to syphilis, and not associated with secondary symptoms, being radically cured. Since these have been distinguished from real syphilis, there have been differences in the treatment of them, and they have been radically cured.

Doubts have been raised whether real syphilis can be radically cured, and those doubts are not new. Mercurialis thought that it was liable, even after the laps of years, to break out again; and the doubts remain in the minds of many whether it can be cured radically or whether it can be cured only temporarily. Well, that doubt may remain until I establish before you that the law regarding syphilis is the same as the law regarding the small-pox, measles, and such like. You can have at the one time only one small-pox, only one cow-pox; and as, just so long as the cow-pox influences the system, you cannot have another small-pox or another cow-pox, so in syphilis; for, as long as the patient is suffering under the syphilitic diathesis arising from an indurated chancre, he cannot have another indurated chancre. The application of this law is that, while a man is suffering under the effects of secondary symptoms, he cannot have a chancre of an indurated character; so that if you want to know whether the system of a man is altogether free from syphilis, you can do so by inoculating him with an indurated chancre; if it takes, he was free; if not, he was insusceptible. That is a great point to be reached in the science of medicine. I say, and say distinctly, that syphilis can be radically cured.

Now as to the case of syphilis in the first stage—the primary sore. You have first to find if this be really the hardened chancre, and it comes with the swelling of the glands; but with it the glands never suppurate. I at once institute the mercurial treatment. Now, there is one point here upon which there is a difference of opinion, for some think that you cannot prevent the secondary symptoms; but I say that if the treatment be well done and soon done—and this is the most important—you can prevent the first bursting out of the secondary symptoms. Why it is not prevented is, that the treatment is applied too late in the first instance, and the secondary often come before the treatment of the primary is commenced. But if you make the treatment of the primary early and effective, the secondary will not appear; I can give you warrant for that. The best treatment for the secondary symptoms is the mercurial, and it must be continued and continuous. In Germany, and in other places as well, the treatment of the secondary symptoms is not continued long enough. You should choose a treatment which which does no harm to the constitution, and continue it for five or six months, and you will have very few cases of relapse; and after the mercurial treatment is finished, go on for another six months with iodine. When a person comes to me, I tell him that he will have to continue under treatment for twelve months. If he will, he will; but if not, then I at once say "good-bye."

But then, you know, there are complications. The treatment I have given you is for syphilis arising in a person who is otherwise healthy, and there is then but one enemy to fight against. But in other cases you may have, in addition, scrofula, or an otherwise bad constitution. Well, then the case is not the same; for many of these constitutional disturbances are interfered with by the syphilitic treatment. In many of these cases the syphilis is the second thing to look at, and you must begin with the constitutional disease first: you must attack the strongest enemy first, and he sometimes waits until you come to him before he opens his attack. Then you must come on gradually with your syphilitic treatment; and that which I prefer in complicated cases is iodide of mercury, which causes little diarrhœa. One capital treatment is that of rubbing in—it is easy and effective. But there are cases in which the rubbing cannot be employed.

In the next stage, I employed iodide of potassium. I use large doses of this, up to 60, 70, 80, and 100 grains a day, and even more. I have made experiments with this; and I have found that, half an hour after the dose has been given, it has passed through the urethra; and it is, in reality, a sort of broom to the blood. The supply must be kept up.

In secondaries, a treatment partially of this iodide and of mercury has its advantages. I have had the potassium stop doing good, and I have gone back to the mercury with good results. That is what Mr. Acton has said, and I quite agree with him.

When syphilis has lasted a long time, and has had great effect upon the constitution, it somehow disappears, and leaves the patient suffering from a complication of diseases which may have been existing before. Well, then you must stop all syphilitic treatment, and repair the deterioration of the blood by iron and bark.

Mr. Acton spoke about the use of bromide of potassium; and I agree with him in its use, for it is a splendid remedy for a complication of syphilis in some cases—in syphilitic diseases of the brain and nervous system; but you cannot depend upon it as an anti-syphilitic remedy.

Now I would impress upon you that you can tell your patients that this terrible disease can be radically cured if they have the courage sufficient to go through the treatment, and their physician have the courage to go through it with them. I again thank you for the cordial reception you have given me.—*British Medical Journal.*—*Nashville Journal of Medicine and Surgery.*

